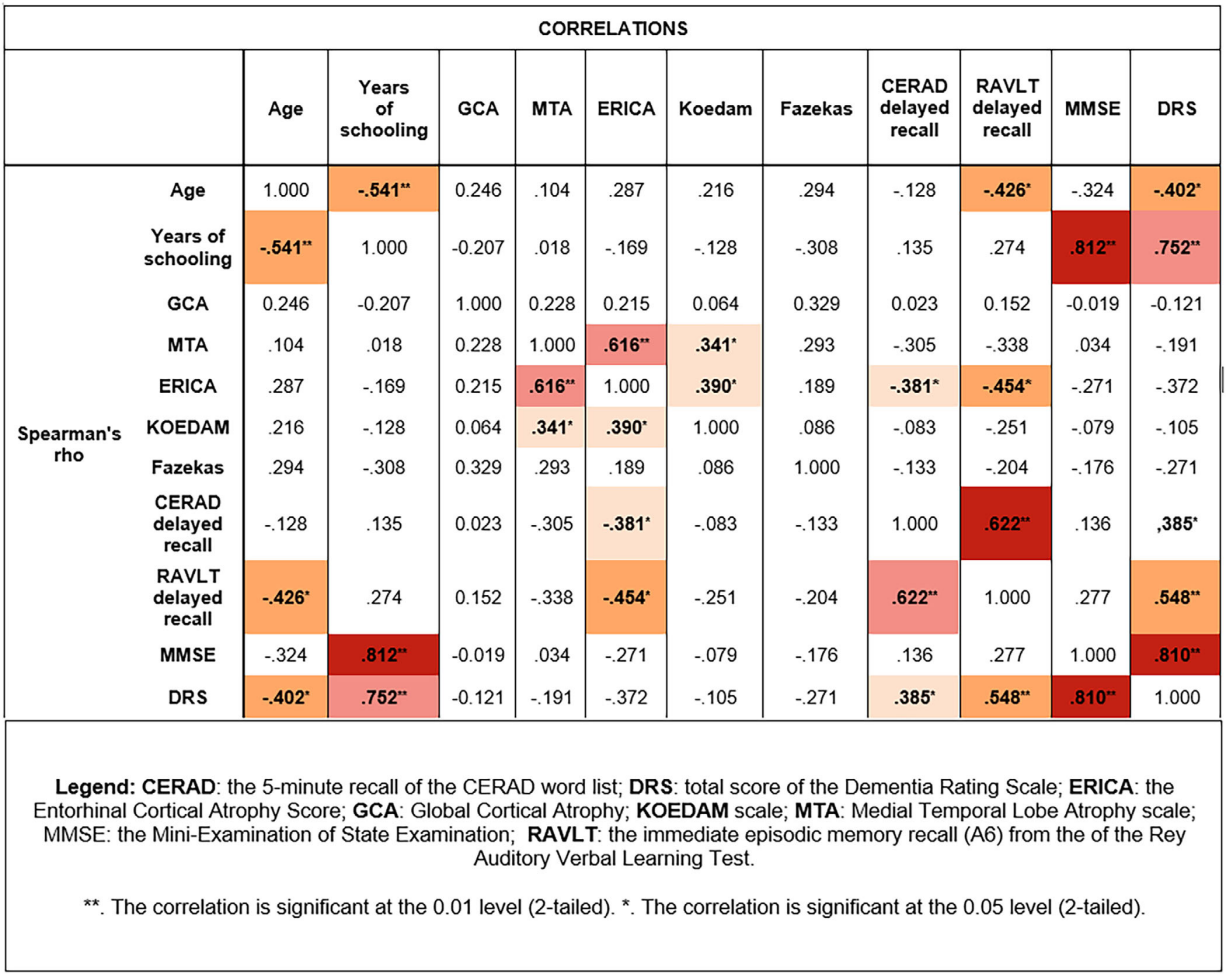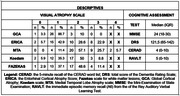# Correlation between MRI Visual Atrophy Scales and cognitive performance in a Brazilian Outpatient Memory Clinic

**DOI:** 10.1002/alz.095070

**Published:** 2025-01-09

**Authors:** Ana Paula Bernardes Real, Gabriela Tomé Oliveira Engelmann, Giovanna Correia Pereira Moro, Carolina Portugal Vieira, Bruna Fugêncio Dias, Thaise Vallesca Queiroz, Leonardo Ryuiti Kimoto, Marcilene Anacleto Melgaço, Aline Siqueira de Souza, Ivonne Carolina Bolaños Burgos, Jonas Jardim de Paula, Marco Aurelio Romano‐Silva, Maria Aparecida Camargos Bicalho, Bernardo M Viana

**Affiliations:** ^1^ Cog‐Aging Research Group, Universidade Federal de Minas Gerais (UFMG), Belo Horizonte, Minas Gerais Brazil; ^2^ Jenny de Andrade Faria Institute – Reference Center for the Elderly, Federal University of Minas Gerais (UFMG), Belo Horizonte, Brazil, Belo Horizonte, Minas Gerais Brazil; ^3^ Hospital das Clínicas da UFMG, University Hospital, Universidade Federal de Minas Gerais (UFMG), Belo Horizonte, Minas Gerais Brazil; ^4^ Older Adult’s Psychiatry and Psychology Extension Program (PROEPSI), School of Medicine, Universidade Federal de Minas Gerais (UFMG), Belo Horizonte, Minas Gerais Brazil; ^5^ Molecular Medicine Postgraduate Program, School of Medicine, Universidade Federal de Minas Gerais (UFMG), Belo Horizonte, Minas Gerais Brazil; ^6^ Undergraduate Medicine, Federal University of Minas Gerais, Belo Horizonte, Minas Gerais Brazil; ^7^ Sciences Applied to Adult Health Postgraduate Program, School of Medicine, Universidade Federal de Minas Gerais (UFMG), Belo Horizonte, Minas Gerais Brazil; ^8^ Department of Psychiatry, School of Medicine, Federal University of Minas Gerais, Belo Horizonte, Minas Gerais Brazil; ^9^ National Institute of Science and Technology Neurotec R (INCT‐MM), Belo Horizonte, Minas Gerais Brazil

## Abstract

**Background:**

Brain imaging plays an important role in Alzheimer’s disease (AD) assessment. Combined with cognitive assessment, MRI helps to exclude other brain disorders and to support AD diagnosis based on the atrophy of specific regions. Visual atrophy scales are useful in clinical practice, especially in low‐income areas where access to biomarkers and automated volumetric analysis are limited.

**Objective:**

To assess the correlation between different MRI Visual Atrophy Scales and cognitive performance in a cohort of a Memory Clinic of Brazilian older adults with heterogeneous educational background.

**Methods:**

Data were collected from the Cog‐Aging Cohort, including participants with normal cognition, mild cognitive impairment, and Alzheimer’s Disease Dementia. Thirty‐five MRI scans were analyzed using the following visual scales: Global Cortical Atrophy (GCA), the Entorhinal Cortical Atrophy Score (ERICA), the Medial Temporal Lobe Atrophy (MTA) scale, and the KOEDAM scale, Fazekas scale for white matter lesions. They were rated by consensus of two memory specialists. These scores were correlated to sociodemographics, to Dementia Rating Scale (DRS), Mini‐Examination of State Examination (MMSE), 5‐minute recall of the CERAD word list, and immediate episodic memory recall (A6) from the of the Rey Auditory Verbal Learning Test (RAVLT).

**Results:**

The mean age of participants was 77.15 years (SD: 74‐80), and the median education was 6.53 years (IQ: 4.48‐8.59). No difference in sex was found. The ERICA scale showed a statistically significant moderate correlation with RAVLT (Rho = ‐.454, p = .017) and a weak correlation with the 5‐minute recall from CERAD’s Word List (Rho = ‐.381, p = .026). No other visual scale demonstrated statistically significant correlations with any cognitive tests. We found strong correlations between the ERICA and MTA scores (Rho = ‐.616, p<.001), and weak but significant correlations between ERICA and KOEDAM (Rho = ‐.390, p = .023).

**Conclusion:**

A higher ERICA score correlated to a worst memory performance. These data corroborate other studies addressing the importance of the use of this scale in research and clinical practice.